# Effect of SrTiO_3_ Nanoparticles in Conductive Polymer on the Thermoelectric Performance for Efficient Thermoelectrics

**DOI:** 10.3390/polym12040777

**Published:** 2020-04-01

**Authors:** Dabin Park, Hyun Ju, Jooheon Kim

**Affiliations:** School of Chemical Engineering & Materials Science, Chung-Ang University, Seoul 06974, Korea; dragoo@naver.com (D.P.); mohani@cau.ac.kr (H.J.)

**Keywords:** thermoelectric, polyaniline, strontium titanate, nanostructure

## Abstract

We present hybrid organic inorganic materials, namely, SrTiO_3_/polyaniline (PANI) composites, with high thermoelectric performance; samples with various SrTiO_3_ contents (10, 20, 30, and 50 wt.%) were prepared. The PANI component was obtained through the polymerization of aniline monomers, followed by camphosulfonic acid-doping to enhance its electrical conductivity. SrTiO_3_, with a high Seebeck coefficient, was used as the N-type inorganic componenet; it was synthesized via a one-pot solvothermal methods and, then, dispersed into the conductive PANI matrix. The SrTiO_3_ content influenced the Seebeck coefficient and electrical conductivity of the resulting composites. The variations in the thermoelectric properties of the SrTiO_3_/PANI composites consequently changed their power factor; at room temperature, the highest value was ~49.6 μW·m/K^2^, which is 17 times larger than that of pure PANI.

## 1. Introduction

Thermoelectric (TE) power generation is a core technology for renewable energy harvesting and greenhouse gas reduction because of the potential energy conversion between thermal and electrical energies [1,2,3,4,5,6,7,8,9]. The efficiency of TE materials can be evaluated by the dimensionless figure of merit, *ZT = S^2^·σ·T/κ*, where *S*, *σ*, *T,* and *κ* are, respectively, the Seebeck coefficient, the electrical conductivity, the absolute temperature, and the total thermal conductivity. The previous studies generally focused on inorganic TE materials such as Te-based compounds (Bi_2_Te_3_, Ag_2_Te, and Cu_2_Te) [10,11,12], Se alloys (SnSe, Cu_2_Se) [13,14,15], and conducting oxides (NaCo_2_O_4_, CaMnO_3_) [16,17]; however, these materials are typically expensive and brittle, which prevents their application in large areas. Thus, polymer-based TE materials have recently been widely used for energy harvesting due to their unique advantages, i.e., low cost, low processing temperature, and mechanical flexibility. In this regard, we have developed high-efficiency metal polymer TE devices [18,19,20].

The *ZT* can be simply achieved by a low thermal conductivity or with a high power factor (*PF = S^2^·σ*). Currently, conductive polymers such as poly(3,4-etylenedioxythiophene)-poly(4-styrenesulfonate) (PEDOT:PSS), polypyrrole, and polythiophene are being used as TE base materials because of their outstanding thermoelectric properties [21,22,23]. Ju et al. enhanced the thermoelectric properties of a conductive PEDOT:PSS matrix by incorporation SnSe nanosheets [24]. SnSe nanosheet/PEDOT:PSS composites have exhibited also enhanced *ZT* (0.32) at room temperature, which is ~6 times larger than that of pristine PEDOT:PSS. Polyaniline (PANI) is another promising conductive polymer due to its unique characteristics, namely, high *σ*, and low *κ* [25,26,27,28]; Anno et al. prepared camphorsulfonic acid (CSA)-doped PANI [29], observing an increased *PF* of ~0.02 μWm/K^2^. Wang et al. observed a *σ* of ~65 S/cm for pure PANI and prepared a PANI/Te nanorod composite via a solution mixing method, achieving high *PF* (~80 μWm/K^2^) at high temperature [30].

Perovskite strontium titanate and its composites are recently being widely used as N-type materials due to their high *S* and chemical stability. In particular, at room temperature, SrTiO_3_ has an outstanding *S* (~400 μV/K) [27] compared to other extensively used N-type materials such as Bi_2_Te_3_, Sb_2_Te_3_, and Ag_2_Te [31,32,33,34].

In this study, we suggest a strategy to increase the thermoelectric performance by fabrication of hybrid polymer-based TE composites, namely, SrTiO_3_/PANI composites. The PANI matrix was synthesized through the polymerization of aniline monomers, followed by the doping with CSA to enhance its *σ*. SrTiO_3_ was prepared via a one-pot solvothermal method by using Sr and Ti precursor solutions. Then, different SrTiO_3_ contents were incorporated into the PANI matrix to obtain hybrid organic-inorganic TE composites, whose thermoelectric properties (S, *σ*, and *PF*) were successively analyzed. We expected that the SrTiO_3_ nanoparticles were randomly dispersed within the PANI matrix, providing a highly efficient thermoelectric performance.

## 2. Experimental

### 2.1. Materials

Sodium hydroxide (NaOH, 98%), ethanol (C_2_H_5_OH, 94%) and hydrochloric acid (HCl, 99%) were purchased from Sigma Aldrich (St. Louis, MO, USA). Strontium nitrate (Sr(NO_3_)_2_, 98%), titanium tetraisopropoxide [(CH_3_)_2_CHO]_4_Ti (TTIP, 98%), ammonium persulfate (APS, 98%), aniline (99%), and D(+)-camphorsulfonic acid(CSA, 98%) were provided by Daejung Chemicals & Metals Co. (Seoul, Korea). All chemicals were used without further purification.

### 2.2. Preparation of PANI

A PANI powder was produced via the chemical polymerization of aniline monomers by using HCl as the dopant and APS as the oxidant. According th the typical synthetic procefure, the monomers (0.05 g) were dispersed in 25 mL of 1M HCl and sonicated the mixture at room temperature. Then, another 1 M HCl solution (1 mL) containing 0.01 g of APS was slowly added to this colloidal solution, followed by steady stirring for 12 h to obtain an aniline/HCL suspension. Next, the polymerization reaction was carried out at 0 °C under stirring for 6 h in an ice bath. The products were filtered and sequentially washed with 1 M HCl, deionized (DI) water, and ethanol, followed by CSA doping to improve the PANI electrical conductivity. The resulting PANI powder was further doped with CSA at a mole ratio of 1:0.6 through a solid-state reaction.

### 2.3. Synthesis of SrTiO_3_

First, Sr(NO_3_)_2_ (1.9 g) and TTIP (2.55 g) were dissolved in DI water (30 mL) and ethanol (180 mL), respectively. The Sr precursor solution was then slowly added to the Ti one, which was subsequently heated and stirred at 50 °C for 30 min. Next, a 0.2 M NaOH aqueous solution (10 mL) was added to the mixture, followed by stirring for another 30 min. Afterwards, the as-obtained solution was poured into a Teflon-lined autoclave and heated at 140 °C for 24 h. The resulting product (SrTiO_3_) was filtered, washed repeatedly, and loaded into a quartz ampoule that was successively sealed with a flame at 350 °C for 30 min.

### 2.4. Preparation of SrTiO_3_/PANI Composites

A quantity of 0.1 g of the CSA-doped PANI and various amounts (10, 20, 30, and 50 wt.%) of SrTiO_3_ were dissolved in m-cresol. These mixtures were stirred for 6 h and filtered twice with DI water and ethanol. Finally, the as-prepared SrTiO_3_/PANI composites were dried in a vacuum oven, ground into fine powders, and hot-pressed at 80 °C for 10 min under 50 MPa.

### 2.5. Characterization of the SrTiO_3_/PANI Composites

The morphology of the samples was examined with a field emission scanning electron microscopy (FE-SEM) system (SIGMA, Oberkochen, German), while their element maps were obtained with an energy dispersive X-ray spectroscopy (EDS) instrument (NORAN system 7, Thermo scientific, Seoul, Korea). An X-ray diffraction (XRD) analyzer (New D8-Advance, Bruker-AXS) with Cu Kα radiation (0.154056 nm) was used to identify the crystal phase of the composites; the XRD patterns were acquired in a 2*θ* range of 20 to 80° at a scan rate of 1°/s. The binding energy peaks were analyzed by using an X-ray photoelectron spectroscopy (XPS) system (Thermo U.K. K-alpha, Seoul, Korea) with Al Kα radiation (1486.6 eV).

Disc-shaped samples with a 12.7 mm diameter were prepared to determine *σ* and *S*. The electrical conductivity was derived by using a Keithley 2400 source meter and the four-point probe method; the thickness of the samples was measured with a digital micrometer. The Seebeck coefficient was estimated with homemade equipment consisting of a pair of voltmeters and thermocouples. Five SrTiO_3_/PANI samples were prepared for the reproducibility of the experiments and the average values are reported here.

## 3. Results and Discussion

In the formation of SrTiO_3_ via the one-pot solvothermal method, NaOH plays a key role as follows. The high OH^−^ concentration provided by the NaOH solution promotes the hydrolysis of TTIP, yielding negatively charged Ti sol that successively reacts with the Sr cations from the Sr precursor, finally forming SrTiO_3_ [31].

**Figure 1:**
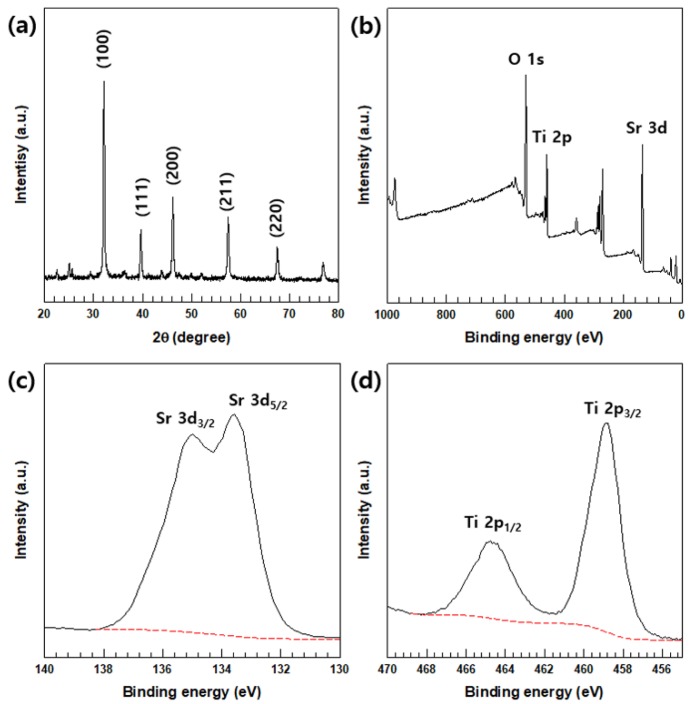
(**a**) XRD patterns, along with the XPS (**b**) survey and high-resolution (**c**) Sr 3d and (**d**) Ti 2p spectra, of the synthesized SrTiO_3_ nanoparticles.

The XRD patterns of synthesized SrTiO_3_ (Figure 1a) showed diffraction peaks at 32.4°, 39.9°, 46.5°, 57.8°, 67.8°, and 77.2°. that were attributed to, respectively, the (100), (111), (200), (211), and (220) planes of perovskite SrTiO_3_ (JCPDS no. 35-0734), indicating the formation of pristine SrTiO_3_ with a well-defined cubic by structure [35,36].

The successful synthesis and chemical composition of SrTiO_3_ was then confirmed by the XPS results. In the XPS survey spectrum (Figure 1b), the Sr 3d and Ti 2p peaks were observed. The high-resolution Sr 3d spectrum (Figure 1c) showed peaks at ~132 and ~134 eV that correspond to the Sr 3d_5/2_ and 3d_3/2_ binding energies, respectively, of pristine SrTiO_3_, suggesting its existence in the Sr^2+^ state. Ti existed as Ti^4+^, as revealed by the two separate peaks at ~463 and ~458 eV, corresponding to Ti 2p_1/2_ and Ti 2p_3/2_, respectively (Figure 1d) [37].

**Figure 2:**
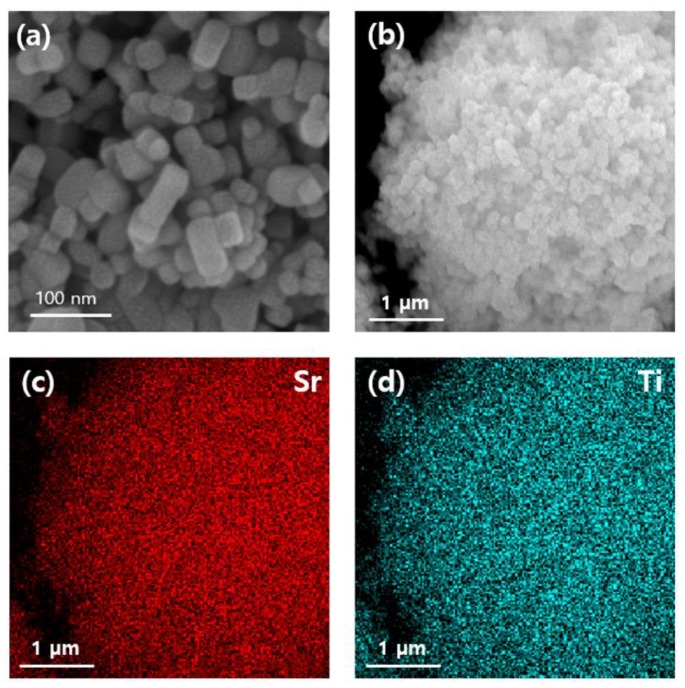
(**a,b**) Field emission scanning electron microscopy (FE-SEM) and energy dispersive X-ray spectroscopy (EDS) images of SrTiO_3_. (**c,d**) Elemental maps of (**b**), obtained via energy dispersive X-ray spectroscopy.

The FE-SEM analysis clearly showed the nano-sized (~50–100 nm) grains of the synthesized SrTiO_3_ (Figure 2a,b). Furthermore, the corresponding Sr and Ti maps (Figure 2c,d) confirmed its unary composition.

**Figure 3:**
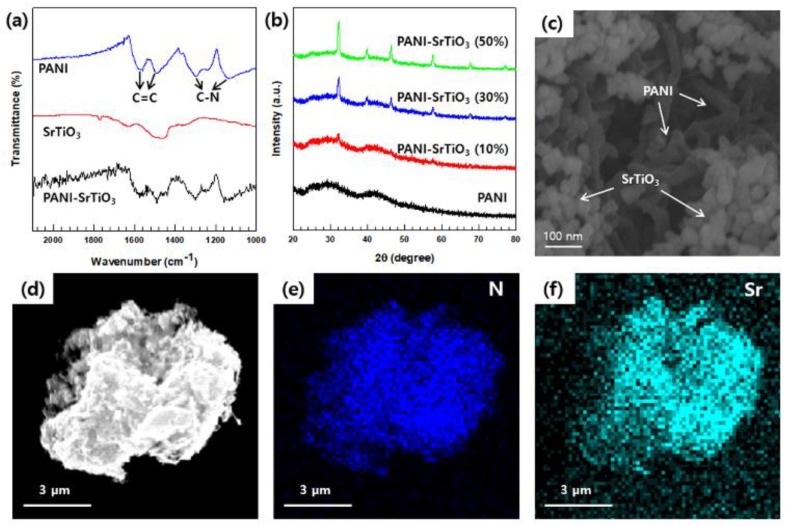
(**a**) FT–IR spectra of the synthesized polyaniline (PANI), SrTiO_3_, and SrTiO_3_ (30 wt.%)/PANI composite. (**b**) X-ray diffraction (XRD) patterns of pure PANI and the SrTiO_3_/PANI composites with different contents SrTiO_3_. (**c**) FE-SEM images and (**d**–**f**) EDS maps of SrTiO_3_(30 wt.%)/PANI.

The FT–IR spectra (Figure 3a) exhibited two bands at at ~1580 and ~1450 cm^−^^1^ and another two at ~1300 and ~1140 cm^−1^; these signal pairs were attributed to the C=C vibration and the C–N structure, respectively, of the quinoid and benzoid rings in PANI [38,39]. The successful preparation of the SrTiO_3_/PANI composites as then confirmed by their XRD patterns (Figure 3b), which showed that the SrTiO_3_ peaks systemically gained intensity as the SrTiO_3_ content increased. These peaks demonstrate the good dispersion of SrTiO_3_ and PANI in the sample composite matrix. The FE-SEM observation of the SrTiO_3_ (30 wt.%)/PANI sample (Figure 3c) further confirmed the uniform dispersion of these two components. Their random dispersion was also demonstrated, along with the successful synthesis of SrTiO_3_/PANI, via the EDS elemental mapping (Figure 3d–f).

For the measurements of the TE properties, the PANI and SrTiO_3_/PANI disks were loaded into a Fe mold and pressed at 200 °C under 50 MPa for 10 min.

**Figure 4:**
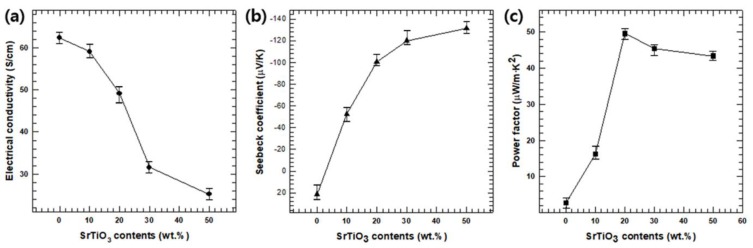
(**a**) Electrical conductivity, (**b**) Seebeck coefficient, and (**c**) Power factor of the SrTiO_3_/PANI composites as functions of the SrTiO_3_ content.

Figure 4a compared electrical conductivities at room temperature of the various SrTiO_3_ /PANI samples and pristine PANI (i.e., 0 wt.%). The pristine PANI sample exhibited a *σ* value of 62.4 S/cm, which is consistent with previous results [26]. As regards the composite sample, *σ* decreased with the increase in the SrTiO_3_ contents; this was due to its relatively lower *σ* of SrTiO_3_ compared to that of PANI [26]. This trend can be explained by the relation of *σ* with the charge carrier mobility (*μ*) and concentration (*n*): *σ = n·e·μ*, where *e* is the electron charge. According to this relation, *σ* is directly proportional to *n* and *μ*, whose values are given in Figure S1. For the SrTiO_3_/PANI samples, *n* decreased when increasing the SrTiO_3_ content, resulting in the above-mentioned *σ* reduction.

The pristine PANI sample exhibited a positive *S* (Figure 4b), confirming its P-type semiconducting behavior. On the other hand, the SrTiO_3_/PANI composites showed negative *S* values due to the N-type semiconducting behavior of SrTiO_3_; furthermore, the absolute *S* value increased along with the SrTiO_3_ content because of the high *S* of SrTiO_3_ at room temperature (~−300 μV/K) [40]. The Sebeck coefficient of a material is generally inversely proportional to *μ* and this trend is described by the following model [41,42]:

(1) $$S=\frac{8\cdot \pi^{2}\cdot k_{B}{}^{2}}{3\cdot e\cdot h^{2}}\cdot m^{*}\cdot T\cdot {\left(\frac{\pi }{3\cdot n}\right)}^{\frac{2}{3}}$$

where *k_B_* is the Boltzmann constant, *h,* is the Planck constant, and m* is the effective mass of the charge carrier.

When increasing the absolute *S* and decreasing *σ*, at room temperature, the SrTiO_3_(20 wt.%)/PANI sample exhibited the largest *PF*, i.e., ~29.6 μW·m/K^2^, which is 17 times larger than that of pristine PANI.

Is this study, we prepared SrTiO_3_/PANI composites and evaluated their thermoelectric properties. With increasing the SrTiO_3_ content, *S* accordingly increased while *σ* decreased. Therefore, the maximum *PF* was obtained for the composite. These experimental results can provide a strategy for achieving highly efficient TE materials.

## 4. Conclusions

We fabricate SrTiO_3_/PANI composites with various SrTiO_3_ contents and analyzed their thermoelectric properties. The purpose was to fabricate PANI-based materials with high thermoelectric efficiency. The PANI and SrTiO_3_ components were synthesized via, respectively, the polymerization of aniline monomers and a one-pot solvothermal method with Sr and Ti precursor solutions; NaOH was crucial in the SrTiO_3_ nanoparticle growth. The morphology and nanostructure of the composites were characterized through XRD, XPS, FT-IR, FE-SEM, and EDS analyses. Upon increasing the SrTiO_3_ content, *σ* decreased because of the low *σ* of SrTiO_3_, while *S* increased. Due to these two trends, at room temperature, the sample with 20 wt.% SrTiO_3_ exhibited the maximum power factor of ~49.6 μW·m/K^2^, which is ~17 times larger than that of pure PANI. The results of this study demonstrate that the combination of SrTiO_3_ with PANI as the organic polymer is a successful strategy to fabricate outstanding organic–inorganic TE materials.

## Supplementary Materials

The following are available online at https://www.mdpi.com/2073-4360/12/4/777/s1, Figure S1: (a) carrier concentration, and (b) Carrier mobility of PANI-SrTiO_3_ composites with various SrTiO_3_ contents at room temperature, Figure S2: (a) FE-SEM images of (a). pristine PANI, (b) pristine SrTiO_3_, and (c) PANI-SrTiO_3_ composites, Table S1. Seebeck coefficient, electrical conductivity, carrier concentration, and carrier mobility of PANI-SrTiO_3_ composites at room temperature.
