# Supplementary material for: Effect of SrTiO3 Nanoparticles in Conductive Polymer on the Thermoelectric Performance for Efficient Thermoelectrics
Source: Polymers (Basel). 2020 Apr 1;12(4):777. doi: 10.3390/polym12040777 (PMC7240522; doi:10.3390/polym12040777)
Supplement: Supplementary file 1 [file polymers-12-00777-s001.pdf]

Supplementary Information

Effect of $SrTiO_3$ Nanoparticles in Conductive Polymer on the Thermoelectric Performance for Efficient Thermoelectrics

Dabin Park, Hyun Ju and Jooheon Kim *

School of Chemical Engineering & Materials Science, Chung-Ang University, Seoul 06974, Korea; dragoo@naver.com (D.P.); mohani@cau.ac.kr (H.J.)

* Correspondence: jooheonkim@cau.ac.kr

Supporting Information Contents:

1. Figures

2. Tables

1. Figures

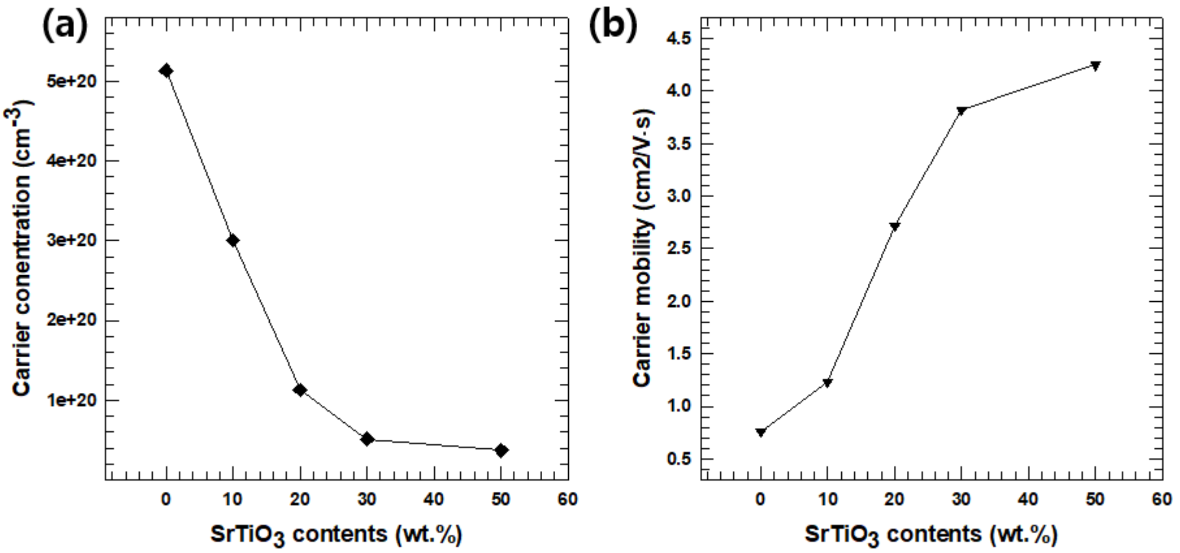

Figure S1. (a) Carrier concentration, and (b) Carrier mobility of PANI-$SrTiO_3$ composites with various $SrTiO_3$ contents at room temperature.

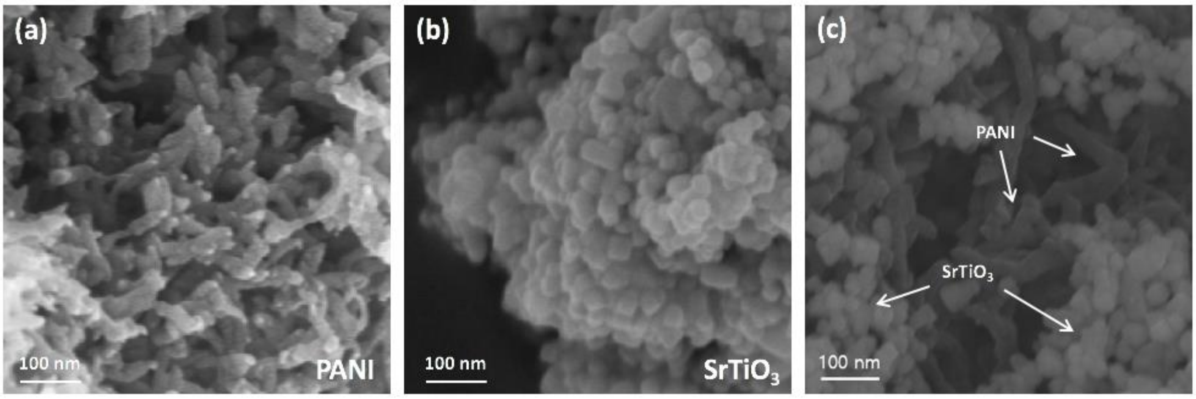

Figure S2. (a) FE-SEM images of (a). pristine PANI, (b). pristine $SrTiO_3$, and (c) PANI-$SrTiO_3$ composites

3. Tables

Table S1. Seebeck coefficient, electrical conductivity, carrier concentration, and carrier mobility of PANI-$SrTiO_3$ composites at room temperature

	S (μV/K)	σ (S/cm)	n (cm^{-3})	μ ($cm^2/V \cdot s$)
PANI	21.24	62.43	5.13×10^{20}	0.76
10 wt.% $SrTiO_3$	-52.45	59.15	3.01×10^{20}	1.23
20 wt.% $SrTiO_3$	-100.42	49.15	1.13×10^{20}	2.72
30 wt.% $SrTiO_3$	-120.12	32.54	5.16×10^{19}	3.82
50 wt.% $SrTiO_3$	-131.32	25.15	3.69×10^{19}	4.25
